# Real-world use of ixekizumab for axial spondyloarthritis treatment in Spain (ESPADA study)

**DOI:** 10.3389/fmed.2025.1704081

**Published:** 2026-01-09

**Authors:** Cristina Campos, Ma Concepción Fito-Manteca, Cristina Valero-Martínez, Sara García-Carazo, Raquel Almodóvar, Silvia Díaz-Cerezo, Sebastián Moyano, Amelia Cobo, Itxaso Aguirregabiria, Clara Pérez-Rambla, Francisco Javier Pérez-Sádaba, Victoria Navarro-Compán

**Affiliations:** 1 Rheumatology Department, General University Hospital of Valencia, Valencia, Spain; 2 Rheumatology Department, University Hospital of Navarra, Navarra, Spain; 3 Rheumatology Department, University Hospital La Princesa, Madrid, Spain; 4 Rheumatology Department, University Hospital La Paz, IdiPaz, Madrid, Spain; 5 Rheumatology Department, University Hospital Fundación Alcorcón, Madrid, Spain; 6 Eli Lilly and Company, Alcobendas, Spain; 7 Outcomes’10 SLU (Product Life Group), Castellón de la Plana, Spain

**Keywords:** axial spondyloarthritis, ixekizumab, treatment persistence, clinical response, real-world study

## Abstract

**Introduction:**

While ixekizumab (IXE) has demonstrated efficacy in axial spondyloarthritis (axSpA) clinical trials, real-world evidence is limited. This study describes the characteristics and treatment persistence of axSpA patients receiving IXE in routine clinical practice in Spain.

**Methods:**

A retrospective study of axSpA patients treated with IXE was carried out in ten hospitals. Demographic, clinical, treatment-related characteristics, persistence and disease activity were collected at baseline, 12, 24 and 52 weeks. Descriptive analysis and Kaplan–Meier methods were used.

**Results:**

The study included 106 axSpA patients, 69.8% had r-axSpA, 58.5% were male, and 63% had overweight or obesity. Mean (SD) disease duration was 12.8 (12.3) years. 98.1% had received b/tsDMARDs and 52.5% presented normal C-reactive protein (CRP) levels at baseline. Persistence rates were 99.0, 80.5, and 56.3% at 12, 24, and 52 weeks, respectively. At week 52, 33.3% fewer patients had high/very high disease activity according to Ankylosing Spondylitis Disease Activity Index (ASDAS-CRP) scores and 18.9% showed an improvement of ≥50% in their Bath Ankylosing Spondylitis Disease Activity Index (BASDAI). Exploratory subanalyses showed IXE persistence was not influenced by gender, smoking status, BMI, axSpA clinical form, CRP levels, HLA-B27 status, prior biological (b) or targeted synthetic (ts) DMARD (b/tsDMARD) or secukinumab. IXE was discontinued by 40 (37.7%) patients during follow-up, mainly due to lack of effectiveness.

**Conclusion:**

Most axSpA patients treated with IXE had long-standing, highly active disease involving multiple domains and prior multiple domains and prior b/tsDMARDs (b/tsDMARD) exposure. Despite this, half remained on IXE at 1 year with improved disease activity, highlighting its potential as a treatment option in daily practice.

## 1 Introduction

Axial spondyloarthritis (axSpA) is a chronic inflammatory rheumatic disease characterized by inflammation and new bone formation in the axial skeleton, including the spine and sacroiliac joints (1, 2). Depending on the presence or absence of definite sacroiliitis as defined by plain X-rays, axSpA is classified as radiographic axSpA (r-axSpA) and non-radiographic axSpA (nr-axSpA) (3). The prevalence of the disease in Spain is in line with other European countries, ranging from 0.3 to 0.8%, with r-axSpA being the most prevalent form (4, 5). Factors influencing the presence of axSpA include being HLA-B27 positive, environmental factors and male sex (4–7). The main musculoskeletal manifestations of axSpA include back pain and progressive spinal rigidity, but it can also affect other domains such as peripheral joints, in the form of arthritis, dactylitis or enthesitis (8). Additionally, the systemic nature of the disease leads to extra-musculoskeletal manifestations including acute anterior uveitis, psoriasis and inflammatory bowel disease (2, 4, 9). Moreover, patients with axSpA often exhibit a higher prevalence of comorbidities associated with their inflammatory status, including higher cardiovascular risk (2, 9, 10).

The pain and reduced mobility associated with the disease has a substantial social, work and emotional burden, negatively affecting overall quality of life (10–13). Consequently, early diagnosis and treatment are essential to maintain functional status and prevent radiographic progression and long-term disability. Recommendations for axSpA management emphasize the importance of individualizing treatment based on the affected domain (axial, peripheral, or extra-musculoskeletal manifestations) as well as their characteristics, including comorbidities and psychosocial factors (14). Current treatment strategies focus on effectively reducing signs and symptoms, and on preventing structural radiographic damage (15, 16). These approaches typically involve patient education, regular exercise, smoking cessation, and physiotherapy alongside the use of nonsteroidal anti-inflammatory drugs (NSAIDs) (14). Use of the latter has reported significant improvements in back pain and stiffness (17), although their prolonged use may be harmful to the gastrointestinal tract and renal and cardiovascular systems (14). Likewise, conventional synthetic disease-modifying antirheumatic drugs (csDMARDs), such as methotrexate and sulfasalazine, have a role in the treatment of peripheral symptoms that coexist with axial disease, although they are not effective for the treatment of axial symptoms. In cases where these drugs are ineffective or not recommended, or if symptoms are predominantly axial, biological and targeted synthetic disease-modifying anti-rheumatic drugs (bDMARDs and tsDMARDs) should be used. The administration of bDMARDs such as tumor necrosis factor inhibitors (TNFi) and interleukin-17 inhibitors (IL-17i) or tsDMARDs such as Janus kinase inhibitors (JAKi) has been recommended as second line treatment in patients with persistently high disease activity (14). In clinical practice, the initiation of a TNFi or IL-17i is the standard approach (14). TNFi have shown efficacy in improving clinical and laboratory variables and reducing magnetic resonance imaging (MRI)-detected inflammation (18–22). However, about 30–40% of patients fail to achieve optimal clinical response (23). Moreover, the management of axSpA is specially challenging due to its long-term nature and bDMARDs loss of efficacy, with up to 50% of patients discontinuing treatment within 4 years (24, 25). Alternative treatment classes targeting interleukin-17, a proinflammatory cytokine participating in neutrophil recruitment, host defense and mediating immuno-inflammation (26, 27), include ixekizumab (IXE). This treatment is currently approved for both r- and nr-axSpA (28) and is available in Spain for axSpA treatment since 2021 (29). Results obtained in COAST clinical trials have demonstrated its efficacy in reducing disease activity and its acceptable safety profile (30), both on bDMARD-naïve and TNFi-experienced patients (31, 32). Although the benefits of IXE treatment have been demonstrated in clinical trials, in real-world clinical practice patient profiles are highly heterogeneous in terms of disease history, previous treatments, and clinical characteristics (30, 33). In particular, there is limited evidence on the effectiveness and persistence of IXE in more complex patient populations, such as those with multiple comorbidities or prior failures to bDMARDs, as well as over extended follow-up periods. Therefore, it is essential to assess treatment persistence and effectiveness in real-life settings (33). In this context, the present study aimed to describe the profile of axSpA patients treated with IXE in Spain, including treatment patterns and disease characteristics, as well as to evaluate the persistence and effectiveness of this treatment in routine clinical practice.

## 2 Methods

### 2.1 Population and study design

**Figure 1:**
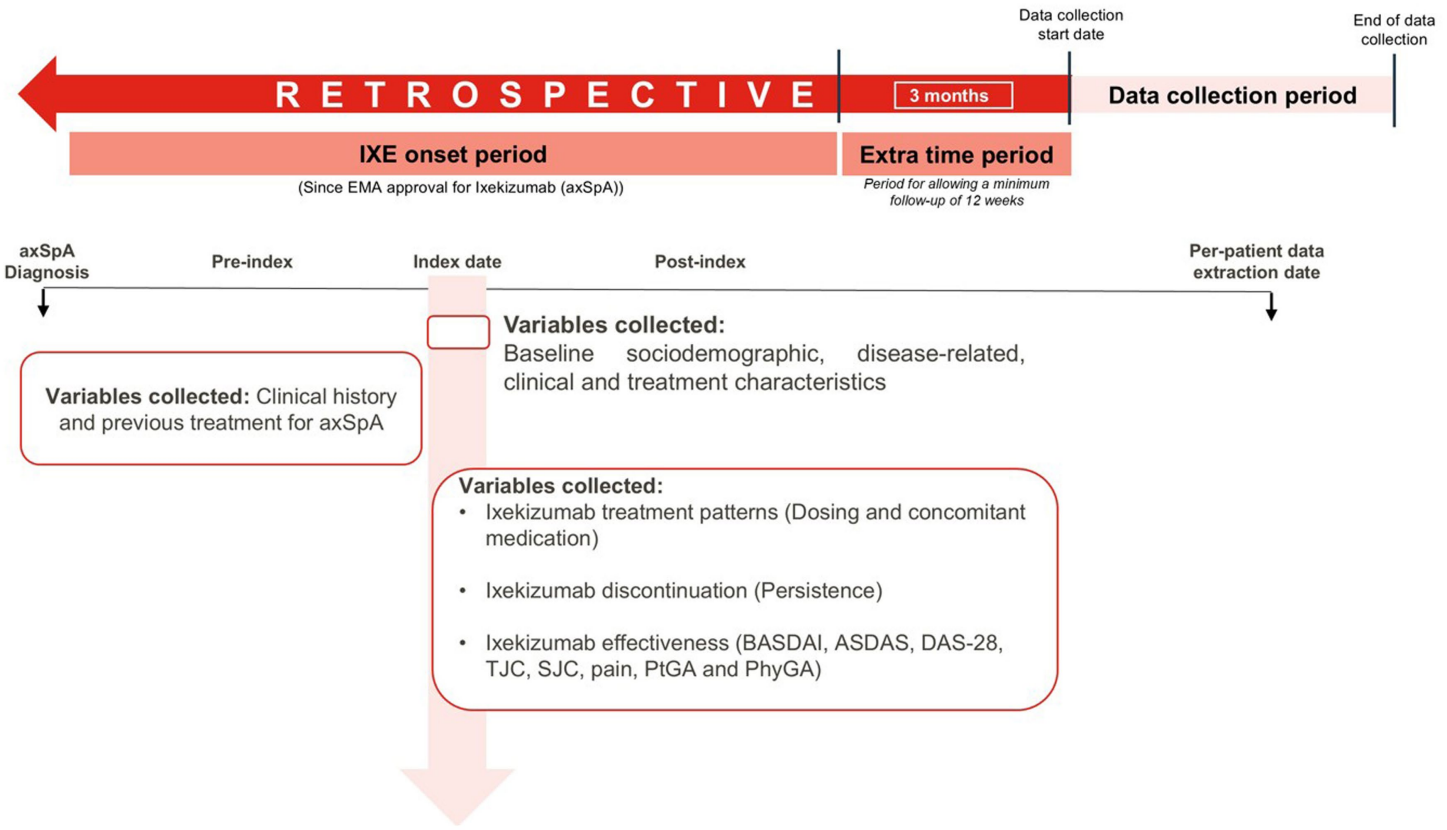
Study design.

ESPADA (use of ixekizumab in patients with axial spondyloarthritis in real-world practice) is a descriptive, multicenter, retrospective observational study conducted in adult patients diagnosed with axSpA and treated with IXE in Spain (Figure 1). The study utilized a medical chart review approach, including data collected between December 2023 and March 2024 across ten Spanish hospitals. As this was a non-comparative observational study, only a single cohort of patients was evaluated.

Patients were included in the study if they met all the following inclusion criteria: (i) over 18 years; (ii) diagnosed with axSpA (r-axSpA or nr-axSpA) according to their rheumatologist and fulfilling the ASAS 2009 classification criteria for axSpA; (iii) had received at least one dose of IXE for axSpA under routine clinical practice; (iv) had initiated IXE treatment between June 1, 2020, and at least 3 months before the starting date of the study, ensuring a minimum follow-up of 12 weeks; and (v) had at least one documented follow-up visit (regardless of continuation with IXE or not).

This study received approval from the Ethics Committee for Drugs Research (CEIm) of the Hospital Universitario La Paz (Reg. 15/2023), and was exempt from the requirement to obtain informed consent from the participants due to its retrospective design. The study complied with the principles outlined in the Declaration of Helsinki, and applicable laws and regulations.

### 2.2 Study objectives and endpoints

The primary objective of the study was to describe the profile of patients with axSpA treated with IXE in routine clinical practice in Spain, including treatment patterns and patient and disease characteristics. Secondary objectives included the analysis of treatment persistence and assessment of clinical response to treatment during follow-up visits (12, 24 and 52 weeks). For this, the percentage of patients who persisted with treatment after 52 weeks and the percentage of patients with improved scores for Bath Ankylosing Spondylitis Disease Activity Index (BASDAI) (34) and Axial Spondyloarthritis Disease Activity Score (ASDAS-CRP) (35) during follow-up visits, respectively, were evaluated.

To gain further insights, exploratory objectives included evaluating treatment persistence across various subgroups, based on gender, smoking status (never, ex- or smoker), BMI (normal weight, overweight or obesity), axSpA type (r-axSpA or nr-axSpA), C-reactive protein (CRP) levels at index date (normal- < 5 mg/L or elevated- ≥ 5 mg/L), presence of HLA-B27 (positive or negative), number of previous biological treatment lines (1 or 2+), and previous secukinumab treatment. Additionally, the clinical response to IXE was explored in patients with concomitant peripheral involvement.

### 2.3 Data collection and study variables

An electronic case report form (eCRF) was used by the physician and the research team. Variables were collected from the patient’s medical records. Neither the sponsor nor the Contract Research Organization in charge of the study had access to any patient identification (ID) data; only the site personnel had access to this information.

For the primary objective, at index date (defined as the day when patients initiated IXE treatment), patients´ variables were collected, including sociodemographic characteristics, disease-related characteristics, clinical characteristics, and treatment patterns (previous treatments, reasons for discontinuation, IXE treatment regimen). For secondary and exploratory objectives aimed at evaluating treatment persistence, date of IXE discontinuation and reasons for discontinuation were collected. In addition, to assess the secondary objective of treatment effectiveness, disease activity indexes of BASDAI and ASDAS-CRP were collected during follow-up visits (12 weeks, 24 weeks, and 52 weeks) when available in medical records. The window period for these visits was ±4, ±6 and ±8 weeks, respectively. Additional disease activity indexes collected were patient’s global assessment of disease activity (PtGA), physician’s global assessment of disease activity (PhyGA) and pain. Furthermore, an exploratory assessment of the clinical response at peripheral level was conducted by collecting disease activity score 28 using CRP levels (DAS-28-CRP), number of tender joints (TJC), and number of swollen joints (SJC).

### 2.4 Instruments and assessments

BASDAI index was calculated using the average of six questions related to fatigue and pain. Each question was scored on a scale of 0 to 10, where scores above 4 were typically indicative of high disease activity. ASDAS-CRP was calculated using an algorithm comprising three BASDAI questions (spinal pain, morning stiffness, and joint pain/swelling), whereby patients were categorized as having inactive disease (ASDAS score <1.3), low activity disease (ASDAS score in range 1.3–2), high activity disease (ASDAS score in range 2.1–3.5) and very high activity disease (ASDAS score >3.5). DAS-28-CRP scores were between 0 and 10, where scores above 5.1 account for high disease activity, and below 3.2 for low disease activity. TJC and SJC were evaluated using a 0–28 or 0–68 and 0–66 scale, respectively.

Pain was evaluated using a 0–10 numeric rating scale where 0 represents “no pain”/ “no affected joints” and 10 “worst pain imaginable”/“10 affected joints.” Finally, PtGA and PhyGA scores were calculated using a 0–10 scale, with higher numbers representing a worse perceived disease activity by the patient or by the physician, respectively.

### 2.5 Statistical analysis

Categorical variables were reported using frequencies (absolute and relative), and the number of patients for each category was included. In contrast, quantitative data was represented as mean and standard deviation (SD). At week 52, the proportion of patients that discontinued IXE treatment was calculated, also detailing the mean time to discontinuation of those patients who discontinued. Discontinuation was considered when a therapy exposure gap exceeded 60 days (36, 37). Finally, persistence was assessed by using the Kaplan–Meier method to estimate the probability of IXE discontinuation at week 12, 24 and 52, censoring patients at the time of their last follow-up. Log-rank tests were used to evaluate differences in persistence between groups.

For the assessment of the clinical response to IXE treatment, all data collected during the follow-up period were considered, irrespective of whether or not patients continued on IXE treatment. Paired comparisons were calculated for each patient with BASDAI, ASDAS-CRP, DAS-28-CRP, TJC, SJC, PtGA, PhyGA, and pain scores with their respective values at the index date. Finally, the mean (SD) change for each index was calculated. A paired *t*-test, Cochran–Mantel–Haenszel test or Wilcoxon signed-rank for paired samples test was employed as required for each case. Statistical analyses were performed using STATA software v.14.

## 3 Results

### 3.1 Sociodemographic, disease-related and clinical characteristics

A total of 106 axSpA patients (69.8% r-axSpA) were included, predominantly men (58.5%) with a mean (SD) age of 51.9 (12.1) years and a disease duration of 12.8 (12.3) years (Tables 1, 2). More than half of patients (63.0%) presented overweight or obesity, and 54.7% of the patients reported a history of smoking. Furthermore, 42.5% of patients experienced comorbidities, the most frequent depression or anxiety (12.3%) (Table 3).

**Table 1:** Sociodemographic characteristics of the study population.

Sociodemographic characteristics (*n* = 106)	
Age, mean (SD) years	51.9 (12.1)
Male, *n* (%)	62 (58.5)
BMI (*n* = 92), *n* (%)	
Underweight (BMI < 18.5)	1 (1.1)
Normal (18.5 ≤ BMI < 25)	33 (35.9)
Overweight (25 ≤ BMI < 30)	33 (35.9)
With obesity (BMI ≥ 30)	25 (27.2)
Smoking habit, *n* (%)	
Ex-smoker	24 (22.6)
Smoker	34 (32.1)
Non-smoker	40 (37.7)
Unknown	8 (7.6)
Employment status, *n* (%)	
Employed	54 (50.9)
Dismissed	7 (6.6)
Retired	14 (13.2)
Not employed	7 (6.6)
Unknown	24 (22.6)
Country of origin, *n* (%)	
Spain	95 (89.6)
Other	11 (10.4)

BMI, body mass index; SD, standard deviation.

**Table 2:** Disease-related characteristics of the study population.

Disease-related characteristics (*n* = 106)	
Descriptive data of disease and treatment, mean (SD) years [IQR]	
Time since diagnosis	12.8 (12.3) [3.2–20.3]
Time since first symptoms to diagnosis	4.2 (6.28) [0.6–5.0]
Time since first symptoms to the start of ixekizumab treatment	16.7 (13.8) [6.5–22.5]
Type of axSpA, *n* (%)	
r-axSpA	74 (69.8)
nr-axSpA	30 (28.3)
Unknown	2 (1.9)
Predominant involvement, *n* (%)	
Axial	73 (68.9)
Peripheral	12 (11.3)
Mixed	21 (19.8)
axSpA features, *n* (%) (multiple response variable)	
Peripheral arthritis	57 (53.8)
Enthesitis	26 (24.5)
Uveitis	14 (13.2)
Dactylitis	9 (8.5)
Psoriasis	30 (28.3)
IBD	3 (2.8)

axSpA, axial spondyloarthritis; IBD, inflammatory bowel disease; IQR, interquartile range; nr-axSpA, non-radiographic axial spondyloarthritis; r-axSpA, radiographic axial spondyloarthritis; SD, standard deviation.

**Table 3:** Clinical characteristics of the study population.

Clinical characteristics (*n* = 106)	
Positive HLA-B27 marker, *n* (%)	55 (51.9)
Family history, *n* (%)	22 (20.8)
Recent MRI, *n* (%)	11 (10.4)
Positive result in MRI (*n* = 11), *n* (%)	9 (81.8)
CRP, mean (SD)	9.3 (12.1)
Elevated CRP (≥5 mg/L) (*n* = 99), *n* (%)	47 (47.5)
ESR, mean (SD)	18.8 (19.8)
Most frequent comorbidities (*n* = 59), *n* (%)	
Depression or anxiety	13 (12.3)
Diabetes	9 (8.5)
Fibromyalgia	8 (7.6)
Peptic ulcer	6 (5.7)
Peripheral vascular disease	4 (3.8)
COPD	4 (3.8)
Any neoplasm	4 (3.8)
Congestive heart failure	3 (2.8)
Rheumatic disease	3 (2.8)
Myocardial infarction	2 (1.9)
Kidney failure	2 (1.9)
Diabetes with complications	1 (0.9)

COPD, chronic obstructive pulmonary disease; CRP, c-reactive protein; ESR, erythrocyte sedimentation rate; HLA-B27, human leukocyte antigen B27; MRI, magnetic resonance; SD, standard deviation.

Regarding laboratory characteristics, 51.9% of patients tested positive for HLA-B27, while 47.5% presented elevated CRP levels at baseline (Table 3). Over half of the patients (53.8%) had previously presented arthritis and 24.5% had enthesitis. Psoriasis was the most frequent extra-musculoskeletal manifestation (28.3%) (Table 2).

At baseline, the mean (SD) BASDAI score was 6.4 (2.0), while over 95.1% of patients were classified as presenting “high disease activity” (60.5%, *n* = 49) or “very high disease activity” (34.6%, *n* = 28) according to ASDAS-CRP (Table 4).

**Table 4:** Disease activity of the study population at index date.

Disease activity at index date	
BASDAI (*n* = 86), mean (SD)	6.4 (2.0)
ASDAS (*n* = 81), *n* (%)	
Very high activity	28 (34.6)
High activity	49 (60.5)
Low activity	3 (3.7)
Inactive	1 (1.2)
DAS-28 (*n* = 21), mean (SD)	3.6 (1.2)
Joint count (*n* = 83), mean (SD)	5.8 (3.3)
PtGA (*n* = 74), mean (SD)	7.2 (1.8)
PhyGA (*n* = 56), mean (SD)	6.2 (1.5)
Present pain (*n* = 99), *n* (%)	99 (93.4)
Back pain	66 (66.7)
Night pain	66 (66.7)
Axial pain	84 (84.8)
Degree of pain (*n* = 99), mean (SD)	
Back pain	7.3 (1.5)
Night pain	7.1 (1.6)
Axial pain	7.2 (1.7)

BASDAI, Bath Ankylosing Spondylitis Disease Activity Index; ASDAS, Ankylosing Spondylitis Disease Activity Score; DAS-28, disease activity score 28; PhyGA, physician’s global assessment of disease activity; PtGA, patient’s global assessment of disease activity; SD, standard deviation.

### 3.2 Treatment patterns

Almost all patients (98.1%) had received at least one treatment line with b/tsDMARDs [mean (SD) number 2.3 (1.4)] prior to IXE (Table 5), mostly TNFi, and 64.2% of patients had received ≥2 b/tsDMARDs. The main reasons for discontinuation of previous treatments were loss of response to treatment/secondary failure (49.8% of bDMARD-treated patients, and 60.0% of tsDMARD-treated patients), followed by lack of response/primary failure (30.9% of bDMARD-treated patients, and 20.0% of tsDMARD-treated patients), and adverse events (13.6% of bDMARD-treated patients, and 0.0% of tsDMARD-treated patients).

**Table 5:** Treatment patterns.

Variables	*n* (%)
Previous treatment lines (*n* = 106)	
0	0 (0)
1	18 (17.0)
≥2	88 (83.0)
Previous b/tsDMARDs (*n* = 106)	
0	2 (1.9)
1	36 (34.0)
≥2	68 (64.2)
Previous treatments (*n* = 106)	
csDMARDs	41 (38.7)
b/tsDMARDs	104 (98.1)
TNFi	100 (94.3)
JAKi	42 (39.6)
Secukinumab	5 (4.7)
Initial ixekizumab dose (*n* = 106)	
80 mg	26 (24.5)
160 mg	80 (75.5)
Monotherapy^a^	59 (55.7)
Concomitant treatments (*n* = 62)^b^	
NSAIDs	54 (87.1)
Glucocorticoids	18 (29.0)
csDMARDs	29 (46.8)

b/tsDMARDs, biologic/targeted synthetic disease-modifying anti-rheumatic drugs; csDMARDs, conventional disease-modifying anti-rheumatic drugs; NSAIDs, nonsteroidal anti-inflammatory drugs.

a Monotherapy described as IXE alone or in combination with an NSAID.

b Percentages calculated based on the total number of patients receiving ixekizumab in combination with other treatments (*n* = 62).

Regarding IXE treatment, many patients (55.7%, *n* = 59) were treated with IXE as monotherapy, defined as IXE alone or in combination with an NSAID (Table 5). Among those who received concomitant csDMARDs, the most frequently prescribed medication was methotrexate (72.4%, *n* = 21). At baseline, 54 (87.1%) patients were receiving NSAIDs. Moreover, 18 (29.0%) patients were receiving glucocorticoids as concomitant treatment. During follow-up, 7 (38.9%) and 11 (16.7%) patients reduced their glucocorticoid or NSAID dose, respectively, while 5 (27.8%) and 7 (13.0%) patients suppressed these concomitant treatments. Details about these treatment modifications are provided in Supplementary Table 1.

### 3.3 Persistence of ixekizumab treatment

**Figure 2:**
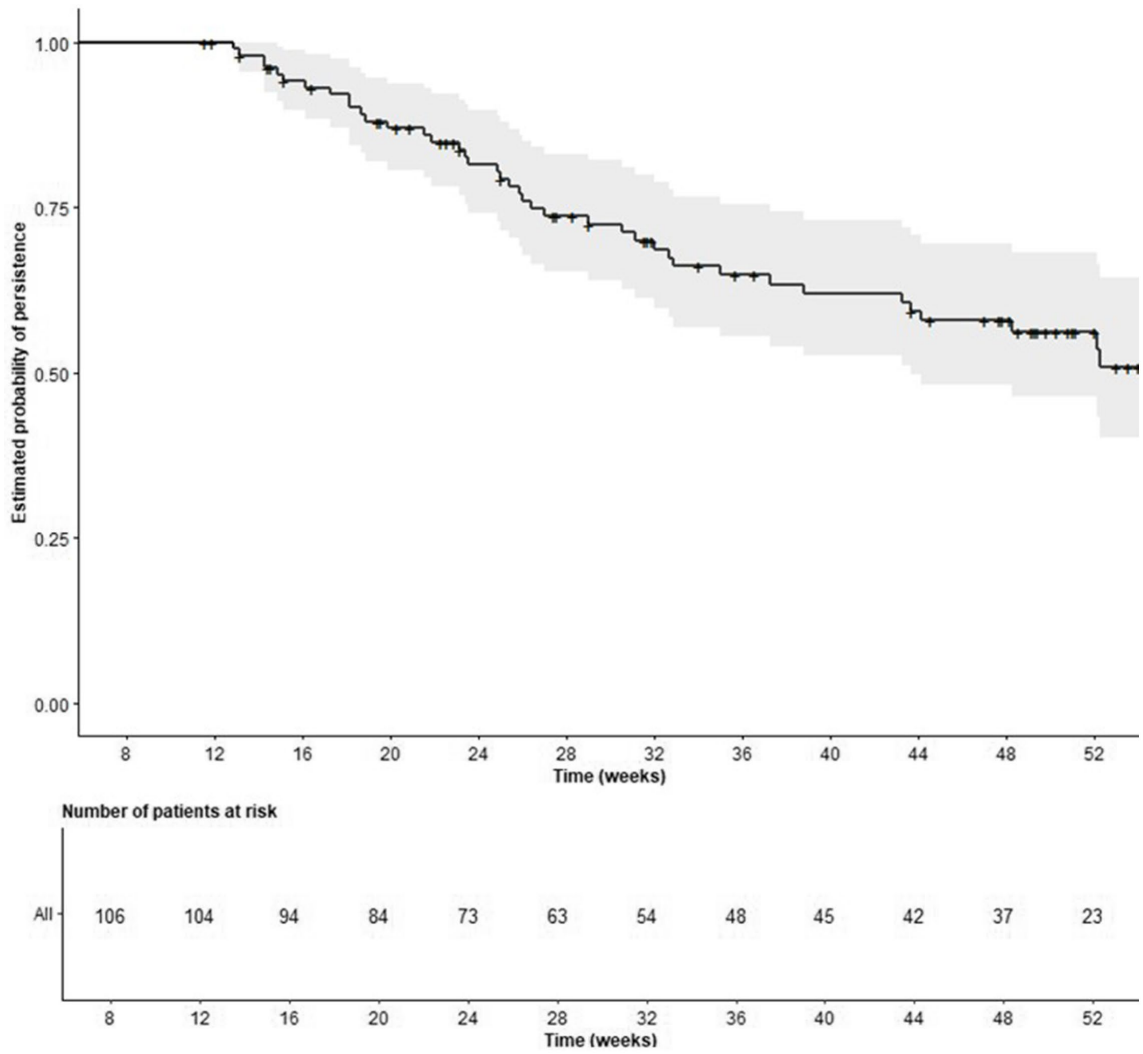
Persistence of ixekizumab. Kaplan–Meier curve.

IXE was discontinued by 40 patients (37.7%) during the 52-week follow-up, with a mean (SD) time to discontinuation of 27.2 (10.9) weeks in those patients who discontinued. IXE persistence showed rates of 99.0, 80.5 and 56.3% at 12, 24 and 52 weeks, respectively, not reaching the median time to discontinuation (Figure 2). The most frequently reported reason for discontinuation was primary non-response (52.5%, *n* = 21), followed by adverse events (22.5%, *n* = 9), and secondary loss of response (20.0%, *n* = 8). The mean time to discontinuation of subgroup analyses is described in Supplementary Table 2.

**Figure 3:**
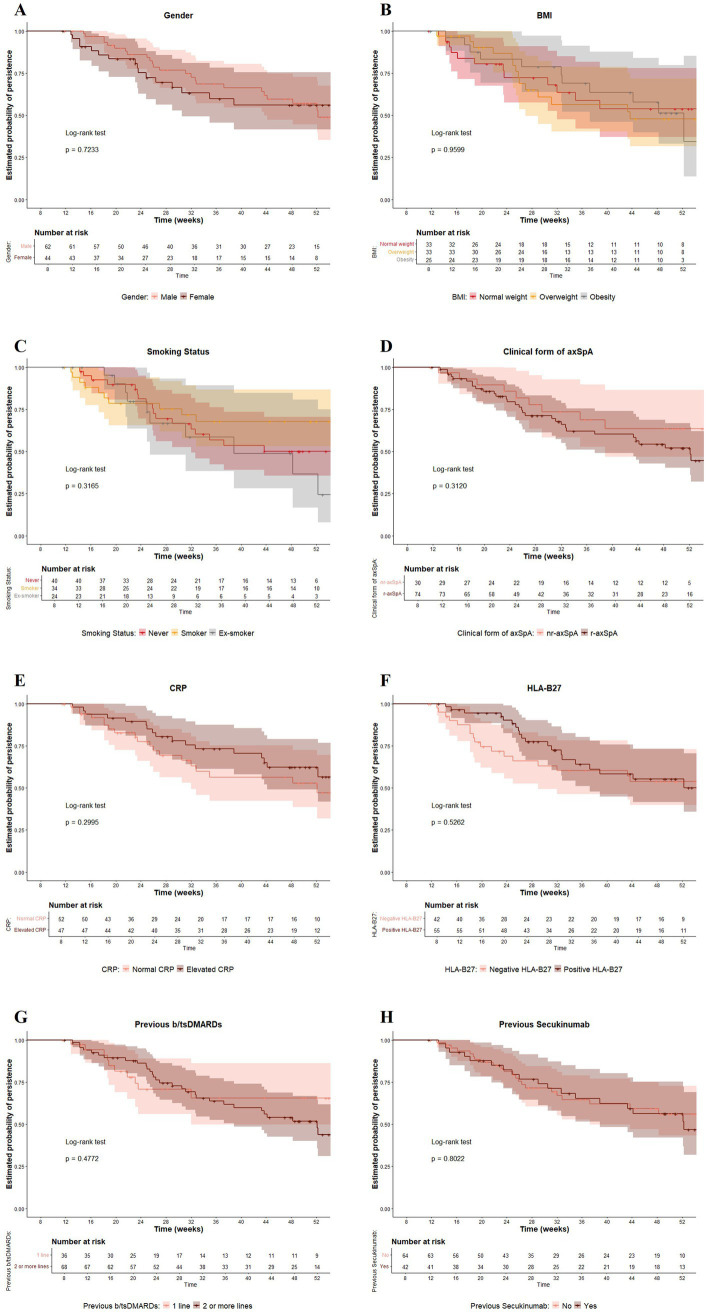
Persistence of ixekizumab treatment by subgroups **(A)** gender, **(B)** BMI, **(C)** smoking status, **(D)** clinical form of axSpA, **(E)** baseline CRP levels, **(F)** HLA-B27 status, **(G)** number of previous b/tsDMARDs, **(H)** previous secukinumab treatment.

Exploratory analyses showed that although persistence appeared slightly higher in certain subgroups, such as patients with nr-axSpA compared to r-axSpA (63.5% vs. 51.9%), those receiving IXE as a second-line b/tsDMARD versus later lines (65.6% vs. 51.8%), patients with normal weight compared to those with overweight or obesity (53.8% vs. 47.7, and 34.3%, respectively), and current smokers compared to never or former smokers (67.8% vs. 50.1, and 24.4%, respectively), none of these differences reached statistical significance. No statistically significant differences in persistence were also found according to gender (male: 56.9%; female: 56.0%, *p* = 0.7), baseline CRP levels (normal: 52.8%; elevated: 47.0%, *p* = 0.3), HLA-B27 status (negative: 53.9%; positive: 50.1%, *p* = 0.5) and previous secukinumab treatment (yes: 56.1%, no: 56.2%, *p* = 0.8) (Figure 3).

### 3.4 Clinical response to ixekizumab treatment

**Figure 4:**
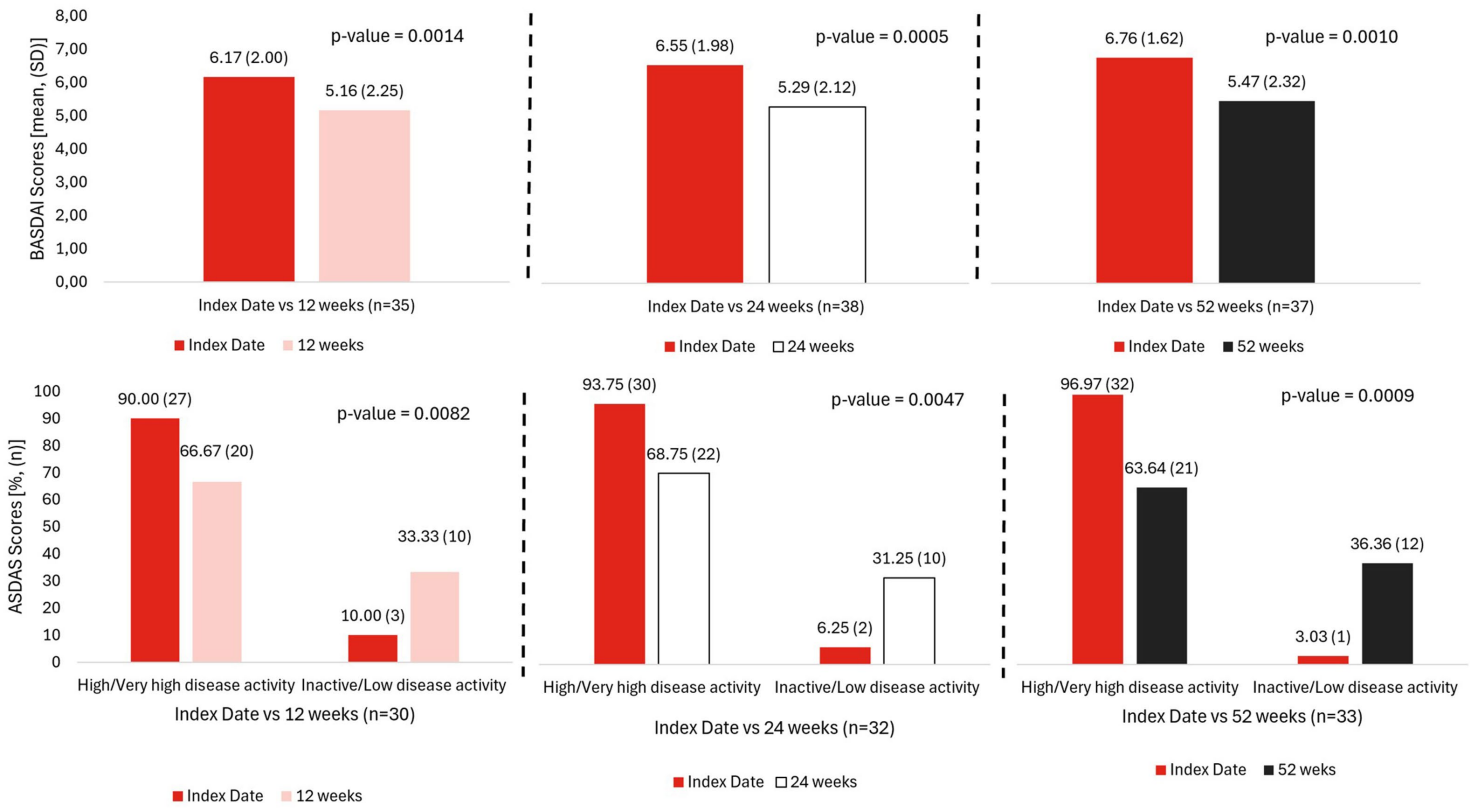
Changes in BASDAI and ASDAS indexes during follow-up. *The number of patients (*n*) at each visit is shown for the available data for this visit.

Among patients with treatment effectiveness data available at week 52, the proportion of patients classified as having “high” or “very high” disease activity by ASDAS-CRP significantly decreased after 12 weeks (66.7%, 20 out of 27 patients, *p* = 0.0082), 24 weeks (68.8%, 22 out of 30 patients, *p* = 0.0047), and 52 weeks (63.6%, 21 out of 32 patients, *p* = 0.0009). Moreover, 18.9% (*n* = 37) achieved BASDAI50. All changes in BASDAI scores were statistically significant, with a mean reduction of −1.0 (*p* = 0.0014), −1.3 (*p* = 0.0005), and −1.3 (*p* = 0.0010) at weeks 12, 24, and 52, respectively (Table 6, Figure 4). Differences in the scores of the additional disease activity indexes between index date and follow-up visits were further analyzed, although the reduction in the number of patients assessed during follow-up should be considered (Supplementary Table 3). A progressive decrease in PhyGA and PtGA indexes was observed at weeks 12, 24 and 52. Changes in PtGA were statistically significant at all timepoints (−1.6, *p* = 0.0000; −0.9, *p* = 0.0139; and −2.1, *p* = 0.0006, respectively), while a significant decrease was observed in PhyGA at week 12 and 24 (−1.5, *p* = 0.0015; −1.3, *p* = 0.0066, respectively), but was not statistically significant at week 52 (−2.3, *p* = 0.0531). This improvement was also noted by a significant reduction in back pain (−1.7, *p* = 0.0001; −1.6, *p* = 0.0055; and −2.0, *p* = 0.004, respectively), pain at night (−1.6, *p* = 0.0002; −1.7, *p* = 0.0027; and −2.2, *p* = 0.0026, respectively), and axial pain (−1.2, *p* = 0.0003; −1.1 *p* = 0.0059; and −1.9, p = 0.0026, respectively). Although slight downward trends were observed during follow-up in other activity indices (DAS-28, TJC, and SJC) among patients with peripheral involvement, these results did not reach statistical significance. (Table 6).

**Table 6:** Changes in disease activity indexes scores during follow-up.

BASDAI		Mean (SD) change	*p*-value*
BASDAI scores	Index date vs. 12 weeks (*n* = 35)	−1.0 (1.7)	0.0014
	Index date vs. 24 weeks (*n* = 38)	−1.3 (2.0)	0.0005
	Index date vs. 52 weeks (*n* = 37)	−1.3 (2.2)	0.0010

ASDAS		High/very high disease activity	Inactive/low disease activity	*p*-value**
ASDAS scores [*n*, (%)]	Index date (*n* = 30)	27 (90.0)	3 (10.0)	0.0082
	Week 12 (*n* = 30)	20 (66.7)	10 (33.3)	
	Index date (*n* = 32)	30 (93.8)	2 (6.3)	0.0047
	Week 24 (*n* = 32)	22 (68.8)	10 (31.3)	
	Index date (*n* = 33)	32 (97.0)	1 (3.0)	0.0009
	Week 52 (*n* = 33)	21 (63.6)	12 (36.4)	

Peripheral involvement		Mean (SD) change	*p*-value***
DAS-28	Index date vs. 12 weeks (*n* = 4)	−1.2 (1.0)	0.0679
	Index date vs. 24 weeks (*n* = 8)	−1.2 (0.9)	0.0117
	Index date vs. 52 weeks (*n* = 3)	−2.3 (1.5)	0.1088
TJC	Index date vs. 12 weeks (*n* = 26)	−0.8 (3.6)	0.1973
	Index date vs. 24 weeks (*n* = 21)	−1.5 (4.8)	0.2028
	Index date vs. 52 weeks (*n* = 10)	−2.3 (3.5)	0.1188
SJC	Index date vs. 12 weeks (*n* = 26)	−0.2 (1.4)	0.3473
	Index date vs. 24 weeks (*n* = 21)	−0.5 (2.4)	0.4611
	Index date vs. 52 weeks (*n* = 10)	−1.3 (2.2)	0.1229

BASDAI, Bath Ankylosing Spondylitis Disease Activity Index; ASDAS, Ankylosing Spondylitis Disease Activity Score; DAS-28, disease activity score 28; SJC, swollen joint count; TJC, tender joint count.

*n* = number of patients at the follow-up date; *Student *t*-test; patients with both visits for comparison were considered; **Cochran–Mantel–Haenszel test; ***Wilcoxon signed-rank for paired samples.

Only patients with both visits were considered for comparison. For SJC and TJC, any scale was allowed; investigators selected the one used (0–28, 0–66, 0–68).

## 4 Discussion

The ESPADA study is a retrospective observational study that characterizes the sociodemographic and clinical characteristics of axSpA patients treated with IXE in Spain, while also offering insights into treatment persistence and clinical response in routine practice. The heterogeneity of the study population, in terms of gender, disease distribution and other factors, enhances the generalizability of the findings to real-world settings.

Our cohort exhibited a more balanced gender distribution compared to clinical trials (31, 38–40), which predominantly include male patients (36), and approximately two-thirds (63.0%) of patients presented with overweight or obesity. This is particularly relevant given the documented sex-related differences in disease expression and management strategies (37, 41), with female patients often exhibiting slower improvements in disease activity (37). The demographic characteristics of our cohort are comparable to those in the BIOBADASER registry, which includes patients with a mean age of 54.2 years and 46.6% males representation (42). These similarities are noteworthy, as BIOBADASER has monitored the safety and effectiveness of biological treatments in Spanish patients since 2000. Our findings are also consistent with those of a Spanish study evaluating another IL-17 inhibitor (43).

Regarding disease presentation, most patients (69.8%) had r-axSpA, while the remaining 28.3% had nr-axSpA, aligning with other real-world studies with proportions ranging from 64–87 to 13–27%, respectively (41, 44). The diagnostic delay in our cohort was 4.2 years, with an average time of 16.7 years from symptom onset to IXE initiation. Although this delay is shorter than previously reported in Spain in 2017, where 75% of patients waited more than 6 years, it still highlights the ongoing challenge of timely diagnosis (45). Comorbidities were present in 42.5% of patients, with depression or anxiety affecting 12.3%, a notably higher rate than the 1–5% reported in other studies (32, 38, 39), which may have negatively impacted treatment outcomes.

HLA-B27 positivity was balanced in our cohort, contrasting with clinical trials that typically report significantly higher rates (36, 38, 40). This marker has been associated with increased susceptibility to the therapeutic benefits of other IL-17 inhibitors (46). Moreover, high CRP has been identified as an efficacy predictor for TNFi, whereas normal CRP levels have been associated with reduced treatment response (47). In our cohort, although approximately 50% of patients presented with normal CRP at baseline, clinical response to IXE was favorable across all patients suggesting no influence of CRP on treatment persistence, consistent with previous studies (31, 48).

Regarding disease involvement, one-third of the patients presented mixed axial and peripheral predominance, and approximately 50% experienced peripheral arthritis (both rates slightly higher than those reported in previous real-world studies) (49–52). Psoriasis was present in approximately 30% of patients, a proportion comparable to that reported by COAST-V-W study (31), but lower than other clinical trials. Uveitis was present in 13.2% of patients, a manifestation with variable prevalence in the literature (44, 53). A low proportion of patients (2.8%) had IBD consistent with the aforementioned studies. Additionally, 95.1% of patients were classified as having “high” or “very high” activity according to ASDAS-CRP at baseline.

In terms of treatment patterns, nearly all patients had received at least one previous therapy with bDMARDs, mostly TNFi, with 64.1% having been treated with two or more prior b/tsDMARDs. This reflects Spanish reimbursement conditions, whereby IXE is covered for patients who have received TNFi or when TNFi are contraindicated (29). This is relevant as clinical trials evidenced that the effects of IXE are conditioned to previous treatments, with naïve patients showing better results compared to TNFi-treated patients. Moreover, extended treatment periods due to consecutive therapies have been associated with increased risk of treatment discontinuation (42). Previous results from COAST-W clinical trial evidenced that treatment with IL-17i of TNFi-experienced patients significantly improves disease activity, which was consistent with the results observed with other IL-17i (54, 55). Both TNFi and IL-17i have proven effective for the treatment of axSpA through overlapping yet distinct inflammatory pathways. These differences contribute to their varying effects on disease manifestations such as psoriasis, IBD, or uveitis (56). Milanez et al. (57) demonstrated that TNF blockade does not influence IL-17 axis activity, whereas IL-17a, in addition to its pathological effects, induces several cascades that result in the expression of several cytokines, including TNFα (58). Altogether, these factors may explain the beneficial effects of IL-17 inhibition observed in TNF-experienced patients. This aligns with ASAS–EULAR recommendations supporting a switch in mechanism toward IL-17a inhibition in patients who fail TNFi (14). Given that 94.3% of our cohort had been previously treated with TNFi, our findings further support the beneficial role of IL-17 inhibition in improving disease activity in bDMARD experienced patients.

Notably, 55.7% of patients in our cohort received IXE as monotherapy, a finding consistent with results from clinical trials (31, 36, 39, 40). Furthermore, almost one third (27.4%) received csDMARDs alongside IXE, likely due to peripheral musculoskeletal involvement. These rates exceed those reported in clinical trials (10, 44, 59), but are comparable to findings from a similar real-world study evaluating IXE in psoriatic arthritis (60).

Kaplan Meier analysis estimated that 56.3% of patients remained on IXE after 52 weeks. These persistence rates were observed in a heavily pretreated population, underscoring robust drug survival even among patients with prior therapeutic failures. Other real-world studies have reported persistence rates similar to those observed in our study, further supporting the consistency of IXE use in routine clinical practice (44, 61, 62). Furthermore, comparable outcomes have been observed with other bDMARDs in similar populations (43).

Although our results showed slightly higher persistence among patients receiving IXE as second-line b/tsDMARD, the difference was not statistically significant. These findings are consistent with other RWE studies, suggesting that IXE persistence is not conditioned by previous bDMARD exposure (44), and that lack of treatment response remains the main reason for IL-17i discontinuation (41, 46, 53). In exploratory analyses, treatment persistence was also not significantly influenced by individual patient characteristics such as gender, BMI, smoking status, HLA-B27 status, and prior secukinumab treatment. Notably, persistence also appeared independent of baseline CRP levels, suggesting that IXE may sustain drug survival even in patients with normal CRP levels, where response to other bDMARDs can be more variable. Similarly, Weddell et al. found that adjusting for sex or prior bDMARD exposure did not significantly alter treatment survival outcomes in pooled analyses of IXE and secukinumab users (44).

Clinical response to IXE was demonstrated by significant reductions across all disease activity indexes, indicating consistent clinical improvement. For example, the mean BASDAI score decreased by 1.3 points at week 52 compared to baseline (*p* = 0.0010). These results are in line with clinical trials reporting BASDAI reductions from week 16 (40) through week 52 (31) and beyond (32, 38), as well as with real-world studies demonstrating similar benefits (41). Our findings also suggest a potential benefit of IXE in axSpA patients with peripheral manifestations. This is supported by a previous study showing reductions in ASDAS, BASDAI, PhyGA and PtGA scores after 52 weeks in bDMARDs-naïve nr-axSpA patients treated with IXE (63). Comparable outcomes were observed in the COAST-X study, which examined IXE impact on sacroiliac joints after 16 weeks, and demonstrated a significant reduction in bone erosion and increases in fat lesions and backfill changes, which are associated with improved bone health and reduced inflammation (36).

Several limitations should be acknowledged. First, the retrospective design may lead to missing data, particularly if patients experiencing improvement did not return for follow-up, introducing potential bias. This limitation should be considered when interpreting results from indices with a reduced number of patients during follow-up. Larger prospective studies are warranted to confirm these findings and provide more definitive conclusions. Nonetheless, our design reflects real-world clinical practice. Future studies should also evaluate radiographic progression, on which real-world data is sparse across axSpA. The lack of standardized data collection timing may introduce variability, as some variables were not recorded uniformly within the designated temporal windows. Lastly, subgroup analyses should be interpreted with caution due to the small sample sizes and the exploratory nature of these comparisons, which did not include *a priori* sample size calculations.

## 5 Conclusion

The ESPADA study offers a comprehensive overview of the demographic and clinical characteristics of axSpA patients treated with IXE in Spain, as well as insights into treatment persistence and effectiveness. Our findings highlight that despite the comorbidity burden and the high rate of prior bDMARD use in our cohort, IXE significantly improved clinical outcomes across all patients and timepoints evaluated while also demonstrating favourable persistence. These findings support IXE as a valuable therapeutic option for axSpA, even in treatment-experienced populations with a long-standing and highly active disease. Further research in larger cohorts is warranted to validate these results and explore the impact of individual patient characteristics on treatment outcomes.

## Data Availability

The raw datasets are not publicly available due to privacy and ethical restrictions.

## References

[1] Navarro-Compán V Sepriano A Capelusnik D Baraliakos X. Axial spondyloarthritis. Lancet. (2025) 405:159–72. doi: 10.1016/S0140-6736(24)02263-3, 39798984

[2] Tam LS Gu J Yu D. Pathogenesis of ankylosing spondylitis. Nat Rev Rheumatol. (2010) 6:399–405. doi: 10.1038/nrrheum.2010.79, 20517295

[3] Reumatología SEd. ESPOGUÍA (actualización 2024) y ediciones anteriores (2018, 2015 y 2009 2024. Available online at: https://www.ser.es/guia-de-practica-clinica-para-el-tratamiento-de-la-espondiloartritis-axial-y-la-artritis-psoriasica/ (Accessed July 23, 2025).

[4] de Reumatología. Guía de Práctica Clínica para el Tratamiento de la Espondiloartritis Axial y la Artritis Psoriásica. Madrid: Grupo de trabajo ESPOGUIA (2015).

[5] Stolwijk C van Onna M Boonen A van Tubergen A. Global prevalence of spondyloarthritis: a systematic review and meta-regression analysis. Arthritis Care Res. (2016) 68:1320–31. doi: 10.1002/acr.22831, 26713432

[6] Sieper J Braun J Dougados M Baeten D. Axial spondyloarthritis. Nat Rev Dis Primers. (2015) 1:1–16. doi: 10.1038/nrdp.2015.13, 27188328

[7] Wang R Ward MM. Epidemiology of axial spondyloarthritis: an update. Curr Opin Rheumatol. (2018) 30:137–43. doi: 10.1097/BOR.0000000000000475, 29227352 PMC5811203

[8] Walsh JA Magrey M. Clinical manifestations and diagnosis of axial spondyloarthritis. J Clin Rheumatol. (2021) 27:e547–60. doi: 10.1097/RHU.0000000000001575, 33105312 PMC8612900

[9] Rudwaleit M Van Der Heijde D Landewé R Listing J Akkoc N Brandt J . The development of assessment of spondyloarthritis international society classification criteria for axial spondyloarthritis (part II): validation and final selection. Ann Rheum Dis. (2009) 68:777–83. doi: 10.1136/ard.2009.108233, 19297344

[10] van Echteld I Cieza A Boonen A Stucki G Zochling J Braun J . Identification of the most common problems by patients with ankylosing spondylitis using the international classification of functioning, disability and health. J Rheumatol. (2006) 33:2475–83. 17013999

[11] Merino M Braçe O González-Domínguez A Hidalgo-Vega Á Garrido-Cumbrera M Gratacós J. Social economic costs of ankylosing spondylitis in Spain. Clin Exp Rheumatol. (2021) 39:357–64. doi: 10.55563/clinexprheumatol/lycdc8, 32662412

[12] Martindale J Shukla R Goodacre J. The impact of ankylosing spondylitis/axial spondyloarthritis on work productivity. Best Pract Res Clin Rheumatol. (2015) 29:512–23. doi: 10.1016/j.berh.2015.04.002 26612245

[13] Kotsis K Voulgari PV Drosos AA Carvalho AF Hyphantis T. Health-related quality of life in patients with ankylosing spondylitis: a comprehensive review. Expert Rev Pharmacoecon Outcomes Res. (2014) 14:857–72. doi: 10.1586/14737167.2014.957679, 25193010

[14] Ramiro S Nikiphorou E Sepriano A Ortolan A Webers C Baraliakos X . ASAS-EULAR recommendations for the management of axial spondyloarthritis: 2022 update. Ann Rheum Dis. (2023) 82:19–34. doi: 10.1136/ard-2022-223296, 36270658

[15] Taurog JD Chhabra A Colbert RA. Ankylosing spondylitis and axial spondyloarthritis. N Engl J Med. (2016) 374:2563–74. doi: 10.1056/NEJMra1406182 27355535

[16] Sieper J van der Heijde D. Nonradiographic axial spondyloarthritis: new definition of an old disease? Arthritis Rheum. (2013) 65:543–51. doi: 10.1002/art.37803 23233285

[17] Song I Poddubnyy D Rudwaleit M Sieper J. Benefits and risks of ankylosing spondylitis treatment with nonsteroidal antiinflammatory drugs. Arthritis Rheum. (2008) 58:929–38. doi: 10.1002/art.23275, 18383378

[18] Davis JJC Van Der Heijde D Braun J Dougados M Cush J Clegg DO . Recombinant human tumor necrosis factor receptor (etanercept) for treating ankylosing spondylitis: a randomized, controlled trial. Arthritis Rheum. (2003) 48:3230–6. doi: 10.1002/art.11325, 14613288

[19] van der Heijde D Dijkmans B Geusens P Sieper J DeWoody K Williamson P . Efficacy and safety of infliximab in patients with ankylosing spondylitis: results of a randomized, placebo-controlled trial (ASSERT). Arthritis Rheum. (2005) 52:582–91. doi: 10.1002/art.20852, 15692973

[20] van der Heijde D Kivitz A Schiff MH Sieper J Dijkmans BA Braun J . Efficacy and safety of adalimumab in patients with ankylosing spondylitis: results of a multicenter, randomized, double-blind, placebo-controlled trial. Arthritis Rheum. (2006) 54:2136–46. doi: 10.1002/art.21913, 16802350

[21] Inman RD Davis JJC Heijde DVD Diekman L Sieper J Kim SI . Efficacy and safety of golimumab in patients with ankylosing spondylitis: results of a randomized, double-blind, placebo-controlled, phase III trial. Arthritis Rheum. (2008) 58:3402–12. doi: 10.1002/art.23969 18975305

[22] Landewé R Braun J Deodhar A Dougados M Maksymowych W Mease P . Efficacy of certolizumab pegol on signs and symptoms of axial spondyloarthritis including ankylosing spondylitis: 24-week results of a double-blind randomised placebo-controlled phase 3 study. Ann Rheum Dis. (2014) 73:39–47. doi: 10.1136/annrheumdis-2013-204231, 24013647 PMC3888598

[23] Rios Rodriguez V Poddubnyy D. Tumor necrosis factor-α (TNFα) inhibitors in the treatment of nonradiographic axial spondyloarthritis: current evidence and place in therapy. Ther Adv Musculoskelet Dis. (2017) 9:197–210. doi: 10.1177/1759720X17706454, 28835779 PMC5557185

[24] Souto A Maneiro JR Gomez-Reino JJ. Rate of discontinuation and drug survival of biologic therapies in rheumatoid arthritis: a systematic review and meta-analysis of drug registries and health care databases. Rheumatology. (2016) 55:523–34. doi: 10.1093/rheumatology/kev374, 26490106

[25] Cramer JA Roy A Burrell A Fairchild CJ Fuldeore MJ Ollendorf DA . Medication compliance and persistence: terminology and definitions. Value Health. (2008) 11:44–7. doi: 10.1111/j.1524-4733.2007.00213.x, 18237359

[26] Girolomoni G Mrowietz U Paul C. Psoriasis: rationale for targeting interleukin-17. Br J Dermatol. (2012) 167:717–24. doi: 10.1111/j.1365-2133.2012.11099.x, 22716185

[27] Chyuan I-T Chen J-Y. Role of interleukin-(IL-) 17 in the pathogenesis and targeted therapies in spondyloarthropathies. Mediat Inflamm. (2018) 2018:1–8. doi: 10.1155/2018/2403935, 29670461 PMC5833467

[28] TALTZ®(Ixekizumab): Summary of product characteristics. (2022). Available online at https://www.ema.europa.eu/en/documents/product-information/taltz-epar-product-information_en.pdf (Accessed July 23, 2025).

[29] sanitarios Aedmyp. Informe de Posicionamiento Terapéutico de Ixekizumab (TALTZ®) en Espondiloartritis axial. (2021). Available online at: https://www.aemps.gob.es/informa/informes-de-posicionamiento-terapeutico/informe-de-posicionamiento-terapeutico-de-ixekizumab-taltz-en-espondiloartritis-axial/ (Accessed July 23, 2025).

[30] Harrison SR Marzo-Ortega H. Ixekizumab: an IL-17A inhibitor for the treatment of axial spondylarthritis. Expert Rev Clin Immunol. (2021) 17:1059–71. doi: 10.1080/1744666X.2021.1970534, 34407705

[31] Dougados M Wei JC Landewé R Sieper J Baraliakos X Van den Bosch F . Efficacy and safety of ixekizumab through 52 weeks in two phase 3, randomised, controlled clinical trials in patients with active radiographic axial spondyloarthritis (COAST-V and COAST-W). Ann Rheum Dis. (2020) 79:176–85. doi: 10.1136/annrheumdis-2019-216118, 31685553 PMC7025731

[32] Deodhar A Poddubnyy D Rahman P Ermann J Tomita T Bolce R . Long-term safety and efficacy of ixekizumab in patients with axial spondyloarthritis: 3-year data from the COAST program. J Rheumatol. (2023) 50:1020–8. doi: 10.3899/jrheum.221022, 36792107

[33] Barbosa CD Balp MM Kulich K Kulich K Germain N Germain N . A literature review to explore the link between treatment satisfaction and adherence, compliance, and persistence. Patient Prefer Adherence. (2012) 6:39–48. doi: 10.2147/PPA.S24752 22272068 PMC3262489

[34] Garrett S Jenkinson T Kennedy LG Whitelock H Gaisford P Calin A. A new approach to defining disease status in ankylosing spondylitis: the Bath Ankylosing Spondylitis Disease Activity Index. J Rheumatol. (1994) 21:2286–91. 7699630

[35] Machado P Landewé R Lie E Kvien TK Braun J Baker D . Ankylosing spondylitis disease activity score (ASDAS): defining cut-off values for disease activity states and improvement scores. Ann Rheum Dis. (2011) 70:47–53. doi: 10.1136/ard.2010.138594, 21068095

[36] Maksymowych WP Baraliakos X Lambert RG Landewé R Sandoval D Carlier H . Effects of ixekizumab treatment on structural changes in the sacroiliac joint: MRI assessments at 16 weeks in patients with non-radiographic axial spondyloarthritis. Lancet Rheumatol. (2022) 4:e626–34. doi: 10.1016/S2665-9913(22)00185-0, 38288892

[37] van der Horst-Bruinsma IE de Vlam K Walsh JA Bolce R Hunter T Sandoval D . Baseline characteristics and treatment response to ixekizumab categorised by sex in radiographic and non-radiographic axial spondylarthritis through 52 weeks: data from three phase III randomised controlled trials. Adv Ther. (2022) 39:2806–19. doi: 10.1007/s12325-022-02132-2, 35429281 PMC9123018

[38] Landewé RB Gensler LS Poddubnyy D Rahman P Hojnik M Li X . Continuing versus withdrawing ixekizumab treatment in patients with axial spondyloarthritis who achieved remission: efficacy and safety results from a placebo-controlled, randomised withdrawal study (COAST-Y). Ann Rheum Dis. (2021) 80:1022–30. doi: 10.1136/annrheumdis-2020-219717, 33958326 PMC8292566

[39] Deodhar A Poddubnyy D Pacheco-Tena C Salvarani C Lespessailles E Rahman P . Efficacy and safety of ixekizumab in the treatment of radiographic axial spondyloarthritis: sixteen-week results from a phase III randomized, double-blind, placebo-controlled trial in patients with prior inadequate response to or intolerance of tumor necrosis factor inhibitors. Arthritis Rheumatol. (2019) 71:599–611. doi: 10.1002/art.40753, 30343531 PMC6593790

[40] van der Heijde D Cheng-Chung Wei J Dougados M Mease P Deodhar A Maksymowych WP . Ixekizumab, an interleukin-17A antagonist in the treatment of ankylosing spondylitis or radiographic axial spondyloarthritis in patients previously untreated with biological disease-modifying anti-rheumatic drugs (COAST-V): 16 week results of a phase 3 randomised, double-blind, active-controlled and placebo-controlled trial. Lancet. (2018) 392:2441–51. doi: 10.1016/S0140-6736(18)31946-9, 30360964

[41] García-Dorta A León-Suarez P Peña S Hernández-Díaz M Rodríguez-Lozano C González-Dávila E . Association of gender, diagnosis, and obesity with retention rate of secukinumab in spondyloarthropathies: results form a multicenter real-world study. Front Med (Lausanne). (2021) 8:815881. doi: 10.3389/fmed.2021.815881, 35096907 PMC8792854

[42] López-Medina C Otero-Varela L Sánchez-Alonso F Jovaní V Expósito-Pérez L Melchor-Díaz S . One-year retention rate of ixekizumab in patients with psoriatic arthritis and axial spondyloarthritis: real-world data from the BIOBADASER registry. Reumatol Clín. (2025) 21:501872. doi: 10.1016/j.reuma.2025.501872, 40619204

[43] Moreno-Ramos MJ Sanchez-Piedra C Martínez-González O Rodríguez-Lozano C Pérez-Garcia C Freire M . Real-world effectiveness and treatment retention of secukinumab in patients with psoriatic arthritis and axial spondyloarthritis: a descriptive observational analysis of the Spanish BIOBADASER registry. Rheumatol Ther. (2022) 9:1031–47. doi: 10.1007/s40744-022-00446-9, 35467242 PMC9314517

[44] Weddell J Din NRA Harrison SR Michelena X McGonagle D Barr A . Real-world experience of IL-17Ai drug survival in a large cohort of axial spondyloarthritis and psoriatic arthritis. Rheumatol Adv Pract. (2024) 8:rkae018. doi: 10.1093/rap/rkae018, 38435412 PMC10907062

[45] Garrido Cumbrera M. Atlas de espondiloartritis axial en España: radiografía de la enfermedad. Madrid: Instituto Max Weber (2017).

[46] Dougados M Lucas J Desfleurs E Claudepierre P Goupille P Ruyssen-Witrand A . Factors associated with the retention of secukinumab in patients with axial spondyloarthritis in real-world practice: results from a retrospective study (FORSYA). RMD Open. (2023) 9:e002802. doi: 10.1136/rmdopen-2022-002802, 36921980 PMC10030893

[47] Wang R Dasgupta A Ward MM. Predicting probability of response to tumor necrosis factor inhibitors for individual patients with ankylosing spondylitis. JAMA Netw Open. (2022) 5:e222312. doi: 10.1001/jamanetworkopen.2022.2312, 35289857 PMC8924712

[48] Sengupta R Baraliakos X Machado P Goupille P Sheesh M Ng KJ . POS0785 efficacy of ixekizumab in radiographic axial spondyloarthritis patients with normal and elevated CRP: a pooled analysis of phase III clinical trials. Ann Rheum Dis. (2025) 84:943–4. doi: 10.1016/j.ard.2025.06.144

[49] de Winter JJ Paramarta JE de Jong HM van de Sande MG Baeten DL. Peripheral disease contributes significantly to the level of disease activity in axial spondyloarthritis. RMD Open. (2019) 5:e000802. doi: 10.1136/rmdopen-2018-000802, 30713720 PMC6340525

[50] López-Medina C Dougados M Ruyssen-Witrand A Moltó A. Evaluation of concomitant peripheral arthritis in patients with recent onset axial spondyloarthritis: 5-year results from the DESIR cohort. Arthritis Res Ther. (2019) 21:139. doi: 10.1186/s13075-019-1927-6, 31171034 PMC6554872

[51] Kenyon M Gallagher P Dinneen B O'Shea F McManus R. Distinct clinical outcomes linked to peripheral arthritis and dactylitis in axial spondyloarthritis: findings from a retrospective Irish cohort. Rheumatol Int. (2024) 44:2517–25. doi: 10.1007/s00296-024-05707-0, 39251445 PMC11424673

[52] Marsh K Mac Gearailt C O'Shea F Fitzgerald G. In axial spondyloarthritis current smoking is associated with lower prevalence of uveitis and peripheral arthritis in males, but not females. Joint Bone Spine. (2024) 91:105746. doi: 10.1016/j.jbspin.2024.105746, 38821214

[53] Strunz PP Englbrecht M Risser LM Witte T Froehlich M Schmalzing M . Analysis of the shorter drug survival times for Janus kinase inhibitors and interleukin-17 inhibitors compared with tumor necrosis factor inhibitors in a real-world cohort of axial spondyloarthritis patients—a retrospective analysis from the RHADAR network. Rheumatol Int. (2024) 44:2057–66. doi: 10.1007/s00296-024-05671-9, 39136784 PMC11392998

[54] Baeten D Sieper J Braun J Baraliakos X Dougados M Emery P . Secukinumab, an interleukin-17A inhibitor, in ankylosing spondylitis. N Engl J Med. (2015) 373:2534–48. doi: 10.1056/NEJMoa1505066, 26699169

[55] Blair HA. Secukinumab: a review in ankylosing spondylitis. Drugs. (2019) 79:433–43. doi: 10.1007/s40265-019-01075-3, 30793255 PMC6422944

[56] Lindström U Bengtsson K Olofsson T Di Giuseppe D Glintborg B Forsblad-d'Elia H . Anterior uveitis in patients with spondyloarthritis treated with secukinumab or tumour necrosis factor inhibitors in routine care: does the choice of biological therapy matter? Ann Rheum Dis. (2021) 80:1445–52. doi: 10.1136/annrheumdis-2021-220420, 34130984 PMC8522450

[57] Milanez FM Saad CG Viana VT Moraes JC Périco GV Sampaio-Barros PD . IL-23/Th17 axis is not influenced by TNF-blocking agents in ankylosing spondylitis patients. Arthritis Res Ther. (2016) 18:52. doi: 10.1186/s13075-016-0949-6, 26912133 PMC4765065

[58] Davydova A Kurochkina Y Goncharova V Vorobyeva M Korolev M. The Interleukine-17 cytokine family: role in development and progression of spondyloarthritis, current and potential therapeutic inhibitors. Biomedicine. (2023) 11:1328. doi: 10.3390/biomedicines11051328, 37238999 PMC10216275

[59] Deodhar A van der Heijde D Gensler LS Kim TH Maksymowych WP Østergaard M . Ixekizumab for patients with non-radiographic axial spondyloarthritis (COAST-X): a randomised, placebo-controlled trial. Lancet. (2020) 395:53–64. doi: 10.1016/S0140-6736(19)32971-X, 31813637

[60] Joven B Hernández Sánchez R Pérez-Pampín E Aragón Díez Á Almodóvar R Martínez-Ferrer Á . Persistence and use of ixekizumab in patients with psoriatic arthritis in real-world practice in Spain. The PRO-STIP study. Rheumatol Ther. (2023) 10:1319–33. doi: 10.1007/s40744-023-00584-8, 37481752 PMC10468471

[61] Danve A Vadhariya A Lisse J Cholayil A Bansal N Bello N . Ixekizumab treatment patterns and health care resource utilization among patients with axial Spondyloarthritis: a retrospective United States claims database study. Rheumatol Ther. (2024) 11:1333–45. doi: 10.1007/s40744-024-00710-0, 39162898 PMC11422398

[62] Jensen KY Grøn KL Hetland ML Glintborg B. Effectiveness of ixekizumab in 709 real-world patients with axial spondyloarthritis and psoriatic arthritis: a nationwide cohort study. RMD Open. (2025) 11:e005806. doi: 10.1136/rmdopen-2025-005806, 40701624 PMC12306273

[63] Braun J Kiltz U Deodhar A Tomita T Dougados M Bolce R . Efficacy and safety of ixekizumab treatment in patients with axial spondyloarthritis: 2-year results from COAST. RMD Open. (2022) 8:e002165. doi: 10.1136/rmdopen-2021-002165, 35853675 PMC9301795

