# Supplementary material for: Real-world use of ixekizumab for axial spondyloarthritis treatment in Spain (ESPADA study)
Source: Front Med (Lausanne). 2026 Jan 9;12:1704081. doi: 10.3389/fmed.2025.1704081 (PMC12827647; doi:10.3389/fmed.2025.1704081)
Supplement: Supplementary file 1 [file Table_1.docx]

**Supplementary Table 1.** Changes in the treatment regimen with NSAIDs and glucocorticoids at each follow-up visit compared to the previous one

|  |  | Week 12  n (%) | Week 24  n (%) | Week 52  n (%) |
| --- | --- | --- | --- | --- |
| NSAIDS | Addition of new drug | 3 (3.6) | 3 (4.6) | 1 (2.6) |
|  | Dose increase | 0 (0.0) | 3 (4.6) | 1 (2.6) |
|  | Active ingredient changes | 2 (2.4) | 1 (1.5) | 1 (2.6) |
|  | Dose reduction | 7 (8.4) | 1 (1.5) | 3 (7.8) |
|  | Suppression | 2 (2.4) | 3 (4.6) | 2 (5.9) |
| Glucocorticoids | Addition of new drug | 2 (2.4) | 1 (1.5) | 0 (0.0) |
|  | Dose reduction | 4 (4.8) | 2 (3.1) | 1 (2.6) |
|  | Suppression | 2 (2.4) | 2 (3.1) | 1 (2.6) |
| TOTAL | | 83 | 65 | 38 |
| *Note: this table describes treatment changes compared to patient's previous visit. A patient may change more than once during follow-up.* | | | | |

Supplementary Table 2. Subgroup analyses assessing time to discontinuation and persistence of those patients that discontinued

|  | **Time to discontinuation (mean [SD] weeks), median [95% CI]** | **Persistence** | | | |
| --- | --- | --- | --- | --- | --- |
|  |  | **Mean (SD) weeks** | **Median** [95% CI] | | |
|  |  |  | **12w** | **14w** | **52w** |
| **According to sex** |  |  |  |  |  |
| Male | 30.3 (11.7), 26.5 [21.8- 34.4] | 45.2 (2.1) | 100  [na- na] | 84.1  [75.1- 94.2] | 56.9  [44.5- 72.7] |
| Female | 22.6 (8.0), 23.3 [15.3- 27.7] | 42.9 (2.9) | 97.7  [93.3- 99.7] | 72.3  [59.5- 87.8] | 56.0  [41.5- 75.5] |
| **According to BMI** |  |  |  |  |  |
| Normal weight | 23.0 (9.0), 21.1 14.9-31.9] | 43.0 (3.5) | 96.9 [91.0- 100] | 72.3 [57.6- 90.9] | 53.8 [78.0- 37.1] |
| Overweight | 26.6 (8.6), 25.6 [21.3-29.4] | 42.0 (3.1) | 97.0 [91.3- 100] | 83.1 [70.5- 97.8] | 47.7 [71.7- 31.7] |
| Obesity | 31.2 (13.2), 32.7 [17.9- 45.3] | 43.8 (3.3) | 95.8 [88.2- 100] | 78.8 [63.8- 97.2] | 34.3 [85.3- 13.8] |
| **According to smoking status** |  |  |  |  |  |
| Never | 26.2 (8.1), 25.9 [23.2-32.0] | 42.5 (2.8) | 97.5 [92.8- 100] | 78.2 [65.9- 92.9] | 50.2 [35.6- 70.6] |
| Smoker | 20.0 (7.4), 17.7 [13.5-29.4] | 45.9 (3.2) | 97.0 [91.3- 100] | 75.2 [61.6- 91.7] | 67.8 [53.1- 86.7] |
| Ex-smoker | 30.2 (12.2), 25.2 [20.4-45.2] | 40.2 (3.8) | 100 [100- 100] | 66.8 [47.9- 93.1] | 24.4 [7.9- 75.1] |
| **According to clinical form** |  |  |  |  |  |
| Radiographic axial spondyloarthritis | 27.8 (11.6), 25.4 [20.7- 31.6] | 42.9 (2.1) | 98.6  [96- 100] | 77.8  [68.5- 88.4] | 51.9  [40.4- 66.8] |
| Non-radiographic axial spondyloarthritis | 25.1 (8.5), 25 [16.4- 34.5] | 46.4 (3.2) | 96.6  [92.8- 100] | 81.8  [68.5- 97.6] | 63.5  [46.7- 86.4] |
| **According to CRP value at baseline** |  |  |  |  |  |
| Normal CRP | 25.7 (10.9), 23.4 [18.1- 31.0] | 42.5 (2.7) | 98  [94.2- 100] | 77.5  [75.1- 95.9] | 52.8  [38.6- 72.4] |
| Elevated CRP | 29.5 (11.6), 26.4 [21.6- 38.8] | 46.7 (2.3) | 97.9  [85.8- 99.7] | 87.1  [77.9- 97.3] | 47.0  [31.8- 69.4] |
| **According to HLA-B27 status** |  |  |  |  |  |
| HLA negative | 22.5 (9.5), 18.9 [16.2- 25.0] | 42 (3.0) | 97.5  [92.8- 99.6] | 65.9  [52.4- 82.9] | 53.86  [39.7- 73] |
| HLA positive | 29.2 (9.4), 26.7 [24.9- 32.8] | 44.8 (2.3) | 100  [na- na] | 88.1  [79.6- 97.5] | 50.1  [35.7- 70.3] |
| HLA unknown | 40.8 (16.5), 48.3 [21.9- 52.1] |  |  |  |  |
| **According to b/tsDMARDs treatment (prior ixekizumab)** |  |  |  |  |  |
| 1 line | 20.4 (5.3), 19.4 [16.1- 23.4] | 45.3 (3.3) | 97.1  [91.8- 100] | 70.6  [56- 89] | 65.6  [49.9- 86.2] |
| ≥2 lines | 29.5 (11.4), 26.7 [24.9- 32.8] | 43.5 (2.0) | 98.5  [95.6- 100] | 84.5  [76- 93.8] | 51.8  [40.2- 66.8] |
| **According to previous secukinumab treatment** |  |  |  |  |  |
| No | 25.3 (9.3), 24.3 [18.9- 27.3] | 44.4 (2.3) | 98.4  [95.4- 100] | 79.2  [69.4- 90.5] | 56.1  [43.3- 72.7] |
| Yes | 29.5 (12.6), 27.5 [19.2- 37.7] | 44.1 (2.6) | 97.6  [93- 100] | 79.5  [67.8- 93.3] | 56.2  [42.1- 75.2] |
|  |  |  |  |  |  |

Supplementary Table 3. Additional disease activity evaluation

|  | | **Mean (SD) change** | **p-value***** |
| --- | --- | --- | --- |
| **PtGA** | Index Date vs 12 weeks (n=52) | -1.6 (2.4) | 0.0000 |
|  | Index Date vs 24 weeks (n=42) | -0.9 (2.3) | 0.0139 |
|  | Index Date vs 52 weeks (n=22) | -2.1 (2.5) | 0.0006 |
| **PhyGA** | Index Date vs 12 weeks (n=30) | -1.5 (2.4) | 0.0015 |
|  | Index Date vs 24 weeks (n=29) | -1.3 (2.3) | 0.0066 |
|  | Index Date vs 52 weeks (n=11) | -2.3 (3.4) | 0.0531 |
| **Back pain** | Index Date vs 12 weeks (n=32) | -1.7 (2.1) | 0.0001 |
|  | Index Date vs 24 weeks (n=21) | -1.6 (2.3) | 0.0055 |
|  | Index Date vs 52 weeks (n=14) | -2.0 (2.2) | 0.0040 |
| **Night pain** | Index Date vs 12 weeks (n=31) | -1.6 (2.1) | 0.0002 |
|  | Index Date vs 24 weeks (n=19) | -1.7 (2.2) | 0.0027 |
|  | Index Date vs 52 weeks (n=15) | -2.2 (2.3) | 0.0026 |
| **Axial pain** | Index Date vs 12 weeks (n=46) | -1.2 (2.1) | 0.0003 |
|  | Index Date vs 24 weeks (n=34) | -1.1 (2.2) | 0.0059 |
|  | Index Date vs 52 weeks (n=18) | -1.9 (2.3) | 0.0026 |
| *PhyGA* p*hysician's global assessment of disease activity, PtGA* patient's global assessment of disease activity, *SD* standard deviation  **** Wilcoxon signed-rank for paired samples.*  *Only patients with both visits were considered for comparison.* | | | |
